# Enhanced Retinal Ganglion Cell Survival via Autophagy Activation in a Novel Retinal Ischemia/Reperfusion Rat Model

**DOI:** 10.3390/ijms27021031

**Published:** 2026-01-20

**Authors:** Si Hyung Lee, Jung Woo Han, Su-ah Yoon, Hun Soo Chang, Tae Kwann Park

**Affiliations:** 1 Department of Ophthalmology, College of Medicine, Soonchunhyang University, 31, Suncheonhyang 6-gil, Dongnam-gu, Cheonan 33151, Republic of Korea; sieh12@schmc.ac.kr (S.H.L.); 106236@schmc.ac.kr (J.W.H.); 2 Department of Ophthalmology, Soonchunhyang University Hospital Bucheon, 170, Jomaru-ro, Bucheon 14584, Republic of Korea; yunsu-a@hanmail.net; 3 Department of Interdisciplinary Program in Biomedical Science, Soonchunhyang Graduate School, Soonchunhyang University Hospital Bucheon, Bucheon 14584, Republic of Korea; intron@schmc.ac.kr; 4 Department of Microbiology, Soonchunhyang University College of Medicine, Cheonan 31151, Republic of Korea; 5 Laboratory of Molecular Therapy for Retinal Degeneration, Soonchunhyang University Hospital Bucheon, Soonchunhyang University College of Medicine, Bucheon 14584, Republic of Korea

**Keywords:** autophagy, retinal ischemia/reperfusion, circumlimbal suture, glaucoma

## Abstract

Autophagy is a fundamental catabolic process that degrades and recycles intracellular components, serving as a key survival mechanism in neurons. In glaucomatous optic neuropathy, autophagy has been linked to both protection of retinal ganglion cells (RGCs) and their accelerated loss, yet its precise impact remains unresolved. In this study, we established and validated a straightforward rat model of retinal ischemia/reperfusion (I/R) using double circumlimbal sutures, which reliably produced RGC apoptosis, retinal thinning, and axonal degeneration compared with controls. Early after reperfusion (1–6 h), robust induction of the autophagy marker LC3B was observed, but this activation diminished within 48 h. Other autophagy-related proteins, including ATG4, ATG7, Beclin-1, and p62, followed similar temporal patterns, while components of the mammalian target of rapamycin (mTOR) pathway displayed an inverse time course. Pharmacologic suppression of mTOR with intravitreal rapamycin administered prior to ischemia provided the most significant neuroprotection, whereas post-injury treatment yielded minimal benefit. Collectively, these findings indicate that timely stimulation of autophagy before retinal ischemic injury can enhance RGC survival and may represent a therapeutic potential for glaucoma management.

## 1. Introduction

Autophagy is an evolutionarily conserved mechanism that maintains cellular homeostasis by eliminating and recycling cytoplasmic components, such as misfolded proteins and damaged organelles [1]. In this process, portions of the cytoplasm are enclosed within autophagosomes and subsequently fused with lysosomes, where their contents are broken down and reused [2]. Such a process supports cell survival under metabolic or environmental stress, enabling adaptation to unfavorable conditions. However, excessive or uncontrolled autophagy can be detrimental, resulting in self-digestion and ultimately triggering programmed cell death [3].

In neurons, maintaining an appropriate level of autophagy is crucial, such that dysregulation in this process may be fatal for neuronal health [3]. Retinal ganglion cells (RGCs), the primary neurons transmitting visual information from the eye to the brain, are particularly vulnerable. Injury to RGCs leads to axonal degeneration and, conversely, axonal damage can precipitate RGC loss, ultimately resulting in irreversible vision impairment. Various ocular disorders contribute to RGC death, including diabetic retinopathy [4,5], retinopathy of prematurity [6,7], and glaucoma [8]. Consequently, diverse animal models have been developed to investigate the mechanisms underlying RGC degeneration.

Among several mechanisms involved in RGC death, retinal ischemia/reperfusion (I/R) mimics the retinal vascular occlusion triggered by hypoxic/ischemic events that eventually cause RGC loss. Both retinal hypoperfusion and vascular insufficiency play roles in the development of glaucoma [9]; thus, models of retinal I/R are widely used to study glaucoma.

The role of autophagy in RGC survival has been investigated with the use of various experimental models, with conflicting results. Numerous studies have indicated that autophagy may have neuroprotective effects on RGCs under stressful conditions [10,11,12,13], while others have suggested that autophagy activation may have detrimental effects on RGC survival under glaucomatous insult [14,15,16]. Recently, we showed that there may be time-dependent differences in autophagic flux after ocular hypertensive (OHT) insult in rats and that activating autophagy immediately after the insult may be favorable for RGC survival under OHT conditions [17]. In light of this, we investigated the time-dependent changes in autophagic flux upon the I/R insult, which is considered another important cause of glaucoma development. In this study, we used a novel I/R model based on a double circumlimbal suture technique, modified from the previously established single circumlimbal suture approach for the OHT rodent model. In addition, by modulating autophagy at different time points before and after the insult, we evaluated the optimal treatment time for promoting RGC survival in the I/R rat model.

## 2. Results

### 2.1. Establishment of Retinal I/R Rat Model Featuring Double Circumlimbal Suture

**Figure 1:**
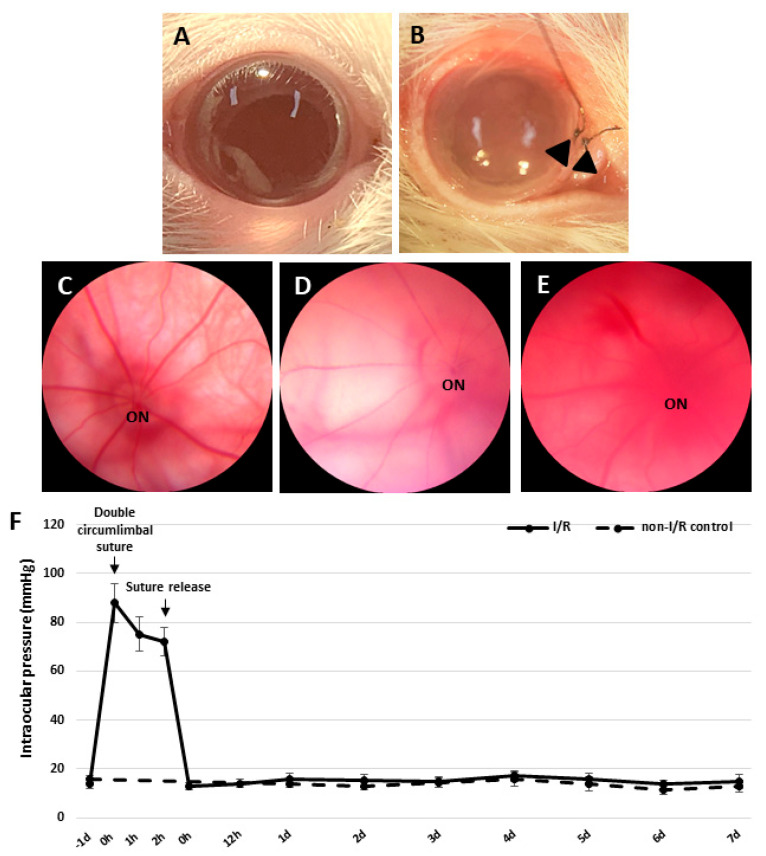
Development of the retinal ischemia/reperfusion model using double circumlimbal sutures. (**A**) The control eye exhibited no gross abnormalities, while double circumlimbal suturing (black arrowheads) rendered the anterior segment pale, suggestive of ischemia (**B**). Fundus photographs showed that retinal circulation was impaired in double circumlimbal sutured eyes (**C**) compared to that of control eyes (**D**). After suture release, reactive hyperemia, possibly of the retina and choroid, was observed, indicating apparent reperfusion (**E**). The intraocular pressure (IOP) was acutely elevated from baseline immediately after the suturing (89 ± 8 mmHg) until the release of sutures; then the IOP fell significantly to 13 ± 2 mmHg and remained within the normal range up to 1 week after surgery (**F**). ON: optic nerve.

Immediately after double circumlimbal suturing, the gross appearance of eyeballs were much paler than that of non-I/R control eyes (Figure 1A,B). Retinal fundus photographs taken before suture placement revealed retinal and choroidal vessels with relatively normal contour and perfusion status (Figure 1C). Double circumlimbal suturing reduced retinal and choroidal circulation, triggering ischemic conditions in the retina and choroid (Figure 1D). Suture release was followed by reperfusion of the retina and choroid; blood flooded back into the vessels (Figure 1E). Such a retinal ischemia/reperfusion event was confirmed by fluorescence angiography, showing a prominent decrease in perfusion of the retina and choroid 2 h after double circumlimbal suturing while suture release allowed rebound reperfusion of the retina and choroid (Supplementary Figure S1). The intraocular pressure (IOP) significantly increased (to 89 ± 8 mmHg) immediately after double circumlimbal suturing and then decreased slightly to 76 ± 7 and 72 ± 6 mmHg 1 and 2 h after the procedure, respectively. After suture release, the IOP fell to 13 ± 2 mmHg and remained in the normal range up to 1 week after surgery (Figure 1F). The IOP of the control eyes was 14 ± 2 mmHg at baseline and 11 ± 1 mmHg at the end of the experiment.

### 2.2. RGC Apoptosis and Decreased Retinal Thickness (RT) After Retinal I/R Insult

**Figure 2:**
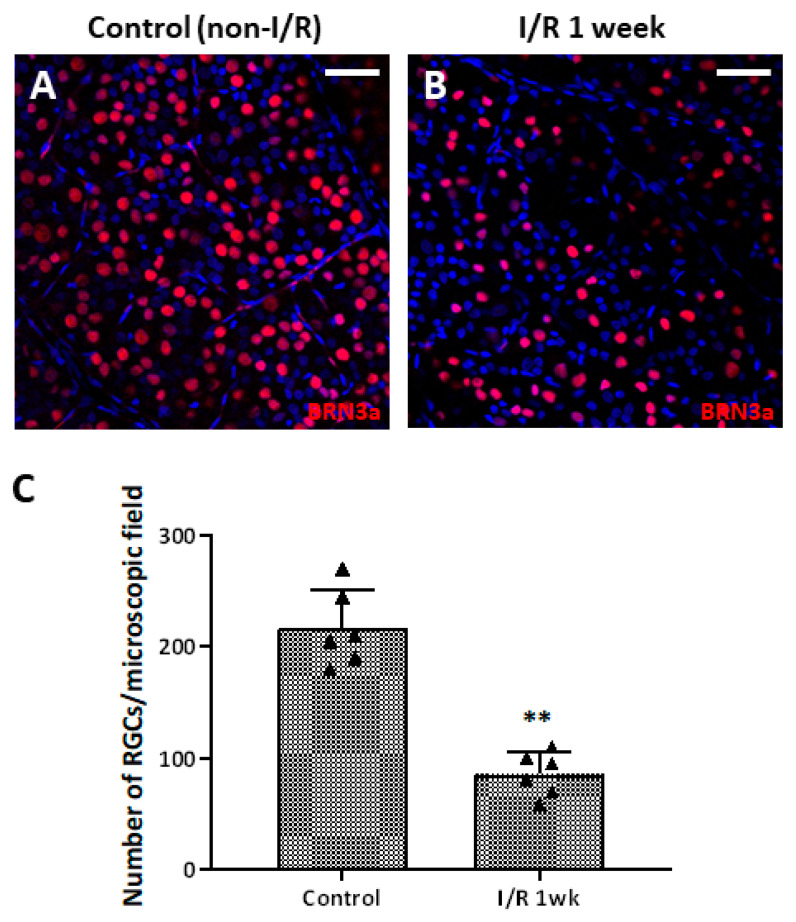
Retinal whole-mount images of control and retinal ischemia/reperfusion (I/R) eyes immunostained for BRN-3a (red) 1 week after double circumlimbal suturing. Immunostaining for BRN-3a 1 week after retinal I/R revealed a decreased number of retinal ganglion cells (RGCs) in retinal I/R eyes (**B**) compared to control eyes (**A**), with a statistically significant difference (**C**). Representative images shown in the figure were photographed from the middle region (2 mm from the optic disc) of the retinal whole-mounts. ** *p* < 0.01. For each group, six biological replicates were included for data analysis (*n* = 6 per group). RGCs were counted in eight peripheral, five mid-region, and three central regions of the whole mounts. The data are means ± standard errors of the means. Scale bar, 40 µm.

**Figure 3:**
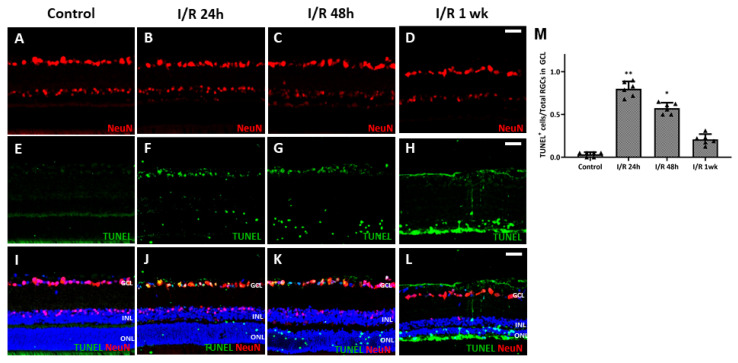
Results from terminal deoxynucleotidyl transferase dUTP nick-end labeling (TUNEL) assay using retinal cross-sections from control and retinal ischemia/reperfusion (I/R) eyes. (**A**,**E**,**I**) No TUNEL-positive retinal ganglion cells (RGCs) were evident in control eyes, but many TUNEL-positive RGCs were apparent 24 h after retinal I/R injury (**B**,**F**,**J**). TUNEL-positive cells were visible up to at least 48 h after the I/R insult with decreasing numbers (**C**,**G**,**K**). One week after the insult, most of the TUNEL-positive cells were in the outer nuclear layer (**D**,**H**,**L**). These changes were significant compared to non-I/R control eyes (**M**). * *p* < 0.05. ** *p* < 0.01. Scale bar, 40 µm. For each group, six biological replicates were included for data analysis (*n* = 6 per group), and the average number of cells from 3 retinal sections for each eye was used for the analysis. Data are means ± standard errors of the means.

To observe whether retinal I/R damaged RGCs, we counted the cell numbers in whole retinal mounts 1 week after I/R insult and compared the results to those of control retinas. RGC numbers fell significantly (compared to the control numbers) 1 week after I/R (Figure 2A–C). To evaluate RGC apoptosis over time after the I/R insult, we performed a dUTP nick-end labeling (TUNEL) assay on retinal cross-sections from controls and I/R rats at 24 and 48 h, and at 1 week after I/R insult. At 24 h, the numbers of TUNEL/NeuN-positive cells had increased markedly compared to the controls; the difference persisted to 48 h. After 1 week, most TUNEL-positive cells were in the outer nuclear layer and the total RT had decreased (Figure 3). We observed a more pronounced reduction in BRN-3a-positive cells in retinal wholemounts compared to NeuN-positive cells in retinal sections, likely because BRN-3a more specifically labels RGC nuclei [18], whereas NeuN stains a broader neuronal population in the GCL, including displaced amacrine cells [19].

**Figure 4:**
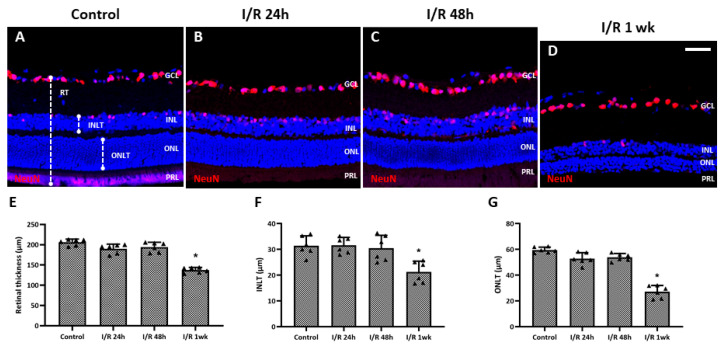
Changes in thickness of the total retina, inner nuclear layer, and outer nuclear layer of eyes after retinal ischemia/reperfusion (I/R) insult. (**A**) Total retinal thickness, inner nuclear layer thickness (INLT), and outer nuclear layer thickness (ONLT) were measured from retinal cross-sections. From cross-sectional images, no apparent changes were detected for retinal thickness (RT) after 24 h (**B**) and 48 h (**C**) after the I/R insult compared to control eyes (**A**), while significant decreases in the total RT, INLT, and ONLT were detected at 1 week after the I/R insult (**D**–**G**). * *p* < 0.05 vs. control. Scale bar, 40 µm. For each group, six biological replicates were included for data analysis (*n* = 6 per group), and the average value from 3 retinal sections for each eye was used for the analysis. Data are means ± standard errors of the means.

The thicknesses of retinal layers were measured and compared using the immunostained retinal cross-sections. Rats 24 and 48 h after insult did not show detectable changes in RT compared to the control (Figure 4A–C). However, a distinct decline in total RT, inner nuclear layer thickness (INLT), and outer nuclear layer thickness (ONLT) was observed at 1 week after I/R insult (Figure 4D) and showed statistically significant differences compared to the control (total RT, *p* = 0.001; INLT, *p* = 0.005; ONLT, *p* < 0.001) (Figure 4D–G).

### 2.3. Ultrastructural Changes in the Optic Nerves After Retinal I/R Insult

**Figure 5:**
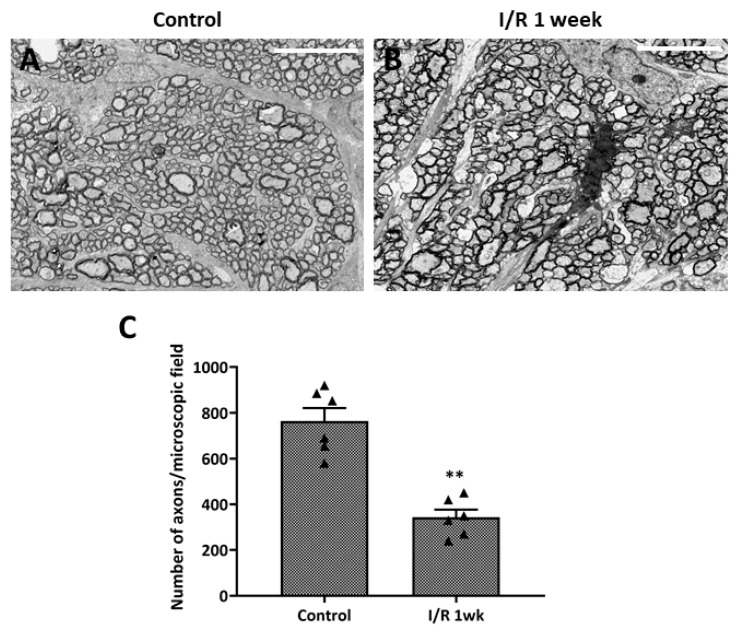
Ultrastructural changes in optic nerve axons after ischemia/reperfusion (I/R) insult. (**A**) Transmission electron microscopy of control eyes revealed no axonal changes or damage of optic nerves, while images from eyes at 1 week after double circumlimbal suturing showed axonal swelling and deformation of the optic nerves (**B**). Statistical analysis showed that the average number of axons per micrograph was significantly lower in retinal I/R eyes than control eyes (**C**). ** *p* < 0.01 vs. control. Scale bar, 10,000 µm. For each group, six biological replicates were included for data analysis (*n* = 6 per group). Data are means ± standard errors of the means.

We evaluated the ultrastructural changes in optic nerve axons under transmission electron microscopy (Figure 5). Control eyes exhibited no axonal damage or density loss. The optic nerves of I/R animals evidenced axonal swelling and deformation. The average axonal count of test rats was significantly lower than that of controls; thus, the I/R insult had induced optic nerve axonal degeneration.

### 2.4. Expression of Autophagy Markers upon Retinal I/R Insult

**Figure 6:**
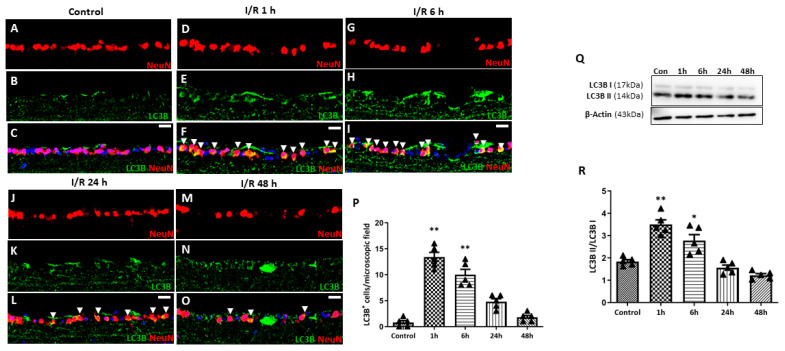
Time-dependent pattern of LC3B expression in eyes with double circumlimbal suture placement. (**A**–**C**) Immunostaining for LC3B showed little to no colocalization with NeuN-positive cells, presumably retinal ganglion cells (RGCs). (**D**–**P**) Markedly increased LC3B expression in RGCs was detected 1 h after reperfusion injury (arrowhead) (**D**–**F**), which gradually decreased over the 48 h period following retinal ischemia/reperfusion (I/R) injury (**G**–**P**). (**Q**,**R**) Quantitative analysis using Western blotting (WB) showed a similar time-course patter:, an initial increase in the LC3-II/LC3-I ratio in the retinas at 1 h after the I/R injury, followed by a gradual decrease over the 48 h period after retinal I/R injury, thus demonstrating early autophagy activation upon retinal I/R damage. Bar graphs are the densitometric values of the protein bands from WB analyses. For each group, five to six biological replicates were included for data analysis (*n* = 5–6 per group). Data are normalized to β-actin and are expressed as mean ± standard errors of the means. Scale bar, 20 µm. * *p* < 0.05. ** *p* < 0.01 vs. control. Statistical significance was determined using Student’s *t*-test.

To evaluate autophagy induction in RGCs, immunohistochemistry (IHC) for microtubule-associated protein light chain 3 (LC3B), an established marker for autophagosome abundance, was conducted (Figure 6). Little to no LC3B expression was detected in the RGCs of control eyes. At 1 h after retinal I/R injury, significantly upregulated expression of LC3B was observed compared to the control, which gradually decreased over the 24 h period following I/R injury, suggesting increased autophagosome formation immediately after retinal I/R. During autophagy induction, LC3-I is converted into LC3-II to stabilize the membrane of the autophagosome. From Western blotting (WB) analysis, we found a similar temporal pattern for the LC3-II/LC3-I ratio upon retinal I/R insult to that observed from IHC staining.

**Figure 7:**
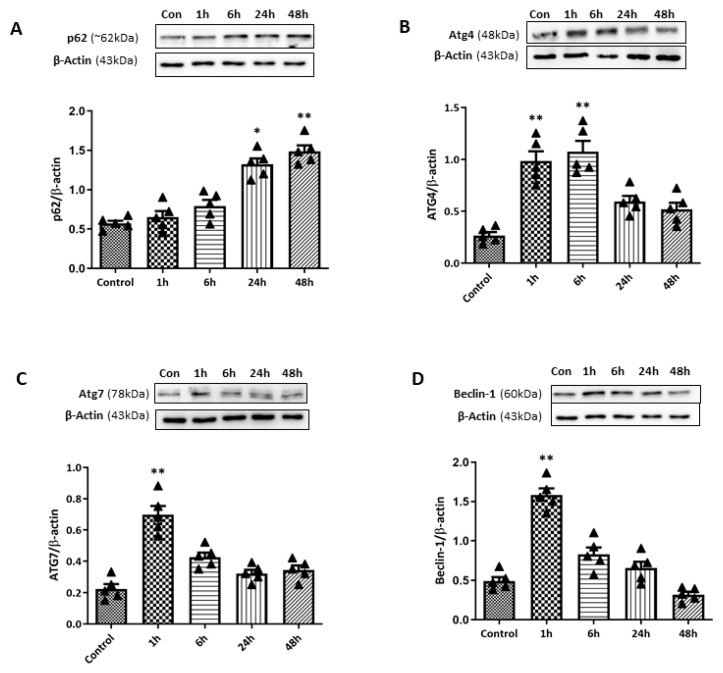
Time-course changes in autophagy markers in retinas after ischemia/reperfusion (I/R) injury using double circumlimbal suture. (**A**) Western blotting (WB) for p62 demonstrated a significant accumulation of the protein 48 h after retinal I/R injury compared to the control retina, indicating autophagy dysregulation at a later period after reperfusion injury. Immunoblotting for ATG family proteins, ATG4 (**B**) and ATG7 (**C**), showed a significant increase in their levels 1 h after double circumlimbal suturing, which decreased to near control levels 24 to 48 h after the injury. Similar findings were observed from WB results for Beclin-1 (**D**). Bar graphs are the densitometric values of the protein bands from WB analyses. For each group, five biological replicates were included for data analysis (*n* = 5 per group). Data are normalized to β-actin and expressed as the mean ± standard errors of the means. Scale bar, 20 µm. * *p* < 0.05. ** *p* < 0.01 vs. control. Statistical significance was determined using Student’s *t*-test.

For further quantification analysis for autophagy-related markers upon retinal I/R insult, WB analyses for various autophagy markers were performed (Figure 7). Ubiquitin-binding protein p62 plays an important role in autophagy by attaching substrates to LC3 for autophagic digestion. While LC3-II levels increased early, p62 levels remained relatively unchanged at 1 and 6 h after retinal I/R insult. Subsequently, p62 levels gradually increased to a significant level at 24 and 48 h after double-circumlimbal suturing. This stable p62 level in the early phase, followed by its accumulation in the later phase despite the initial LC3-II increase, suggests that autophagic flux may become progressively impaired after the initial insult. In addition, expression levels of ATG proteins including ATG4 and ATG 7 were analyzed. ATG4 and ATG7 promote the lipidation and conjugation of LC3 with phosphatidylethanolamine, respectively. Both ATG4 and ATG7 were significantly elevated at early time points and at 1 and 6 h after retinal I/R insult, which decreased to near control levels 24 to 48 h after the injury. ATG6, also known as Beclin-1, plays a crucial role in autophagosome formation. Similar to other ATG proteins, a significant increase in the Beclin-1 level was detected 1 h after the surgery, followed by a gradual decrease up to 48 h, indicating an initial induction of the autophagic machinery early after retinal I/R development.

### 2.5. Changes in the Activation of the mTOR Pathway and Its Correlation with Autophagic Markers in I/R Eyes

**Figure 8:**
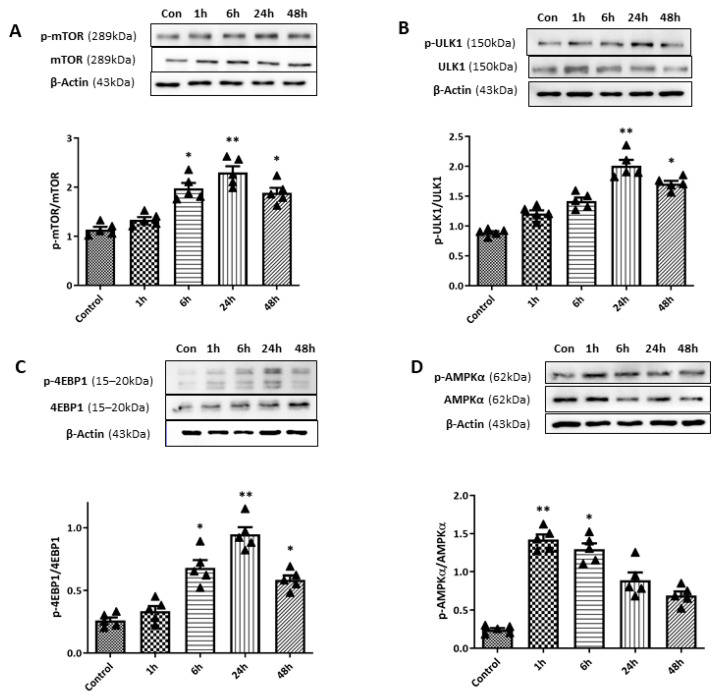
Changes in mammalian target of rapamycin (mTOR) pathway activity upon retinal ischemia/reperfusion (I/R) insult using double circumlimbal suture. (**A**) Western blotting (WB) analyses showed a gradual increase in the phosphorylated mTOR (p-mTOR)/mTOR ratio within 24 h of the surgery, with a statistically significant increase at 6 and 24 h after double circumlimbal suturing. Expression levels of Unc51-like kinase 1 (ULK1) and 4E-binding protein 1 (4EBP1), downstream targets of the mTOR signaling pathway, demonstrated increased p-ULK1/ULK1 (**B**) and p-4EBP1/4EBP1 ratios (**C**) at 6 and 24 h after the surgery; a similar pattern was observed for the p-mTOR/mTOR ratio. Both ratios decreased 4 weeks after the surgery. (**D**) The activity of the AMP-activated kinase (AMPK) pathway (the p-AMPK/AMPK ratio) showed an opposite time-course compared to the mTOR pathway, with an early significant increase at 1 and 6 h, followed by a decrease at 24 and 48 h after retinal I/R injury. Bar graphs are the densitometric values of the protein bands from WB analyses. For each group, five biological replicates were included for data analysis (*n* = 5 per group). Data are expressed as the mean ± standard errors of the means. Scale bar, 20 µm. * *p* < 0.05. ** *p* < 0.01 vs. the control. Statistical significance was determined using Student’s *t*-test.

To examine the relationship between the mTOR pathway and the observed autophagy induction during the early period after retinal I/R, we conducted WB analyses of phosphorylated mTOR (p-mTOR) (Figure 8). A gradual increase in the p-mTOR/mTOR ratio was observed up to 24 h after the retinal I/R insult, reaching statistical significance after 6 (*p* < 0.05), 24 (*p* < 0.01), and 48 h (*p* < 0.01) after the surgery. During autophagy suppression, p-mTOR inhibits Unc51-like kinase 1 (ULK1) and 4E-binding protein 1 (4EBP1) via their phosphorylation; these are two main downstream targets of the mTOR signaling pathway. Therefore, we further assessed p-ULK1 and p-4EBP1 levels. Similar patterns were observed for p-ULK1 and p-4EBP1 levels, which were significantly elevated at 6, 24, and 48 h after the surgery. On the other hand, the expression level of AMP-activated kinase (AMPK), which is inversely correlated with mTOR activity, was also investigated by WB analyses. The phosphorylated AMPK level showed a significant increase 1 h after retinal I/R insult, which decreased over the 48 h period following surgery; thus, the opposite time-dependent pattern was exhibited by phosphorylated AMPK levels compared to that observed for the p-mTOR level.

### 2.6. Enhanced RGC Survival upon Intravitreal Rapamycin Injection via Modulation of Autophagy

**Figure 9:**
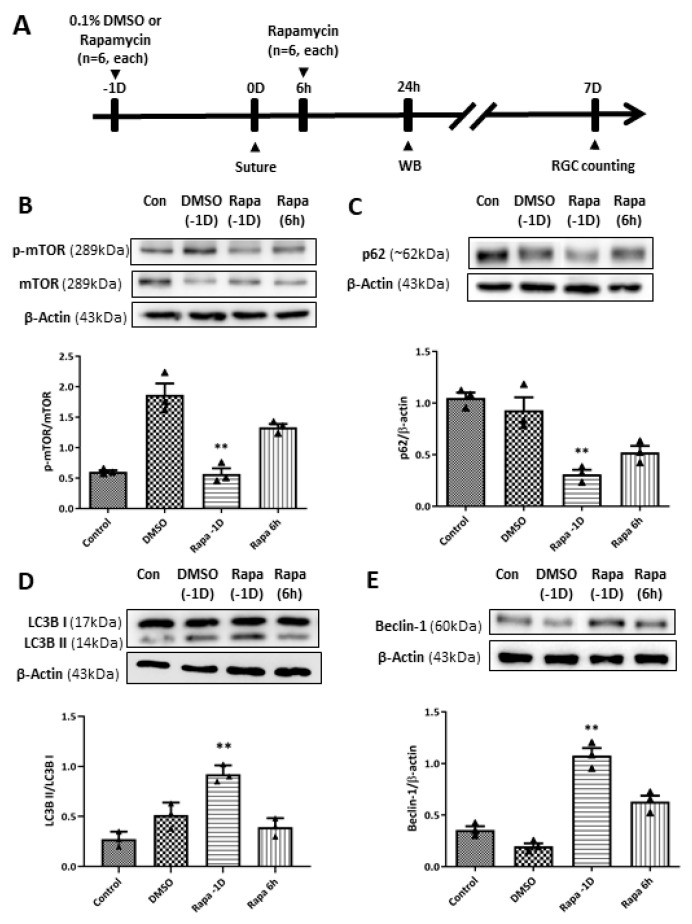
Modulation of the mammalian target of rapamycin (mTOR) pathway and autophagy induction by intravitreal rapamycin injection in eyes with retinal ischemia/reperfusion (I/R) injury. (**A**) Experimental timeline: 0.1% dimethyl sulfoxide (DMSO) or rapamycin was intravitreally injected at 1 day before (–1D) or 6 h after retinal I/R insult. Animals were sacrificed at 24 h or 7 days after reperfusion for Western blotting (WB) analysis or retinal ganglion cell counting, respectively. (**B**) Intravitreal rapamycin injection at –1D resulted in a significant decrease in the p-mTOR/mTOR ratio compared to DMSO-injected eyes, whereas injection at 6 h post-injury showed a slight reduction without statistical significance. Autophagy induction upon rapamycin injection was assessed by (**C**) p62, (**D**) LC3-II/LC3-I ratio, and (**E**) Beclin-1. Significant enhancement of autophagic markers was observed in the –1D rapamycin group, characterized by a decrease in p62 and increases in Beclin-1 and the LC3-II/I ratio compared to the DMSO-injected group. In contrast, rapamycin administered at 6 h post-injury did not reach the same magnitude of autophagy modulation. Bar graphs represent the densitometric values from WB analyses. For each group, three biological replicates were included for data analysis (*n* = 3 per group). Data are normalized to β-actin (except for p-mTOR/mTOR and LC3-II/I ratios) and expressed as the mean ± standard errors of the means. ** *p* < 0.01 vs. the control. Statistical significance was determined using Student’s *t*-test.

To further investigate the effect of autophagy induction on RGC survival in our retinal I/R rat model, we performed intravitreal rapamycin injection at different time points according to the retinal I/R insult: 1 day before (day-1) and 6 h after surgery (Figure 9). Intravitreal 0.1% dimethyl sulfoxide (DMSO) injection was performed on day-1 for the vehicle control group. WB analyses showed that mTOR pathway activity was significantly reduced by intravitreal rapamycin administration when it was given a day before suture placement compared to DMSO-injected retinal I/R eyes. In addition, autophagy induction was significantly upregulated upon intravitreal rapamycin injection given 1 day before surgery compared to intravitreal DMSO-injected eyes, as shown by p62, LC3B, and Beclin-1 expression.

**Figure 10:**
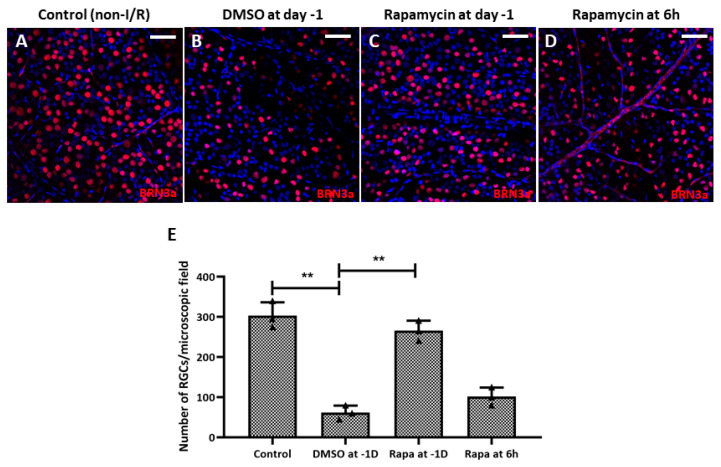
Intravitreal injection of rapamycin at 1 day before double circumlimbal suturing enhanced retinal ganglion cell (RGC) survival. (**A**,**B**,**E**) Whole-mount images immunostained for BRN-3a exhibited a significantly lower number of RGCs in retinal ischemia/reperfusion (I/R) eyes with intravitreal 0.1% dimethyl sulfoxide (DMSO) injection given 1 day before double circumlimbal suture compared to non-I/R eyes. (**C**–**E**) Retinal I/R eyes with intravitreal rapamycin injection performed at day 1 showed a significantly increased number of RGCs compared to eyes with 0.1% DMSO injection. However, eyes with intravitreal rapamycin injection performed 6 h after the insult did not show a significant difference in RGC numbers compared to DMSO-injected eyes. Representative images shown in the figure were photographed from the middle region (2 mm from the optic disc) of the retinal whole-mounts. For each group, three biological replicates were included for data analysis (*n* = 3 per group). RGCs were counted in eight peripheral, five mid-region, and three central regions of the whole mounts. Data are expressed as mean ± standard errors of the means. ** *p* < 0.01. Scale bar, 40 µm.

Regarding RGC survival, we found that intravitreal rapamycin injection 1 day before double-circumlimbal suturing led to a significant increase in RGC numbers in retinal I/R eyes compared to the intravitreal DMSO injection group, whereas intravitreal rapamycin injected 6 h after surgery did not show statistically significant RGC survival effects (Figure 10). Considering that mTOR inhibition triggers autophagy induction, autophagy induction early before I/R development may have potent neuroprotective effects by promoting RGC survival.

## 3. Discussion

We developed a novel retinal I/R model using double circumlimbal suturing; this is a modification of the existing single circumlimbal suture model [20,21]. Immediately after double suturing, the IOP increased to 90–99 mmHg, but fell to within the normal range 3 h after suture release. IHC and TUNEL assay measurements revealed that retinal I/R had been effectively established; transmission electron microscopic images of the optic nerve supported the retinal findings. Using this simple and novel retinal I/R rat model, we further investigated the role of autophagy on RGC survival under retinal I/R conditions and found that autophagic markers (LC3-II and Beclin-1) increased significantly during the early period of retinal I/R, over the 1 to 6 h of reperfusion. However, this initial induction was not sustained, and the subsequent accumulation of p62 suggests that autophagic flux became impaired as the injury progressed. Upregulating autophagy at different times through intravitreal rapamycin injection revealed that rapamycin given before the I/R insult showed the most potent effect on RGC survival compared to that administered after the injury. These findings suggest that increasing autophagic flux before the injury may have neuroprotective potential in acute retinal I/R conditions.

Conventional I/R establishment is an invasive procedure. The cornea is punctured with an infusion needle that is then connected to a bottle of sterile saline to induce acute elevation of the IOP (over 100 mmHg for 1 h) [22]. Although the procedure is straightforward, there is a risk that intraocular structures, such as the iris and lens, may be damaged while puncturing the cornea, and the infusion is sometimes stopped because the needle is unstable. Therefore, a safer, noninvasive, and reproducible means of developing I/R is required. A recent, novel, simple technique induced chronic IOP elevation in an animal model by placing a simple circumferential suture around the globe approximately 1.0 mm behind the limbus [20,21,23,24,25]. The single circumlimbal suture technique is primarily used to induce chronic ocular hypertension (COH), where the intraocular pressure (IOP) gradually falls after an initial spike and is maintained at a moderately elevated level for up to 15 weeks. In contrast, our study required a model that reliably induces acute retinal I/R injury followed by a return to normal IOP. We modified the technique by placing double circumlimbal sutures to create a more effective acute ischemia of the retina and choroid. This modification ensured the IOP was acutely elevated to a higher level (around 89 ± 8 mmHg) for the fixed 2 h duration of ischemia. Subsequent suture release induced reperfusion and allowed the IOP to return to the normal range quickly. The double suture effectively provided the acute I/R insult needed for this study without damaging any intraocular structure, and suture release after 2 h of ischemia triggered RGC apoptosis and retinal thinning 7 days later. The IHC and TUNEL assay data revealed markedly increased apoptosis of both RGCs and inner nuclear cells 24 h after the I/R insult; this continued for at least 48 h. Previous reports using a conventional retinal I/R model also found that retinal I/R initially damaged the inner retina [5,26]. In addition, 1 week after the I/R insult, most TUNEL-positive cells were in the outer nuclear layer, reflecting outer retinal damage developing subsequent to inner retinal injury. The total RT, as well as the thickness of the ONL and the INL, decreased significantly 1 week after the I/R insult, as noted in previous studies using conventional I/R models [26,27].

The role of autophagy in neurodegenerative conditions is complex, often described as having a dual function—being either protective or detrimental—which varies according to the specific pathological neuronal condition. Several studies have suggested that impairments in autophagy may contribute to the development of Alzheimer’s disease [28,29,30], Parkinson’s disease [31,32], and amyotrophic lateral sclerosis [33,34]. However, autophagy can accelerate neurodegeneration in certain circumstances [35,36], leaving room for further research.

Meanwhile, the role of autophagy in the glaucomatous condition has also been reported with conflicting results using various glaucoma animal models, including COH and retinal I/R models. A study that used a COH model induced by episcleral vein cauterization showed that the levels of autophagy markers LC3-II and Beclin-1 were significantly increased at 1 to 2 months after the initial insult; additionally, the results indicated that inhibiting autophagy activity through 3-methyladenine administration has neuroprotective effects [14].

In an earlier study that used the COH model induced by circumlimbal suture, we found similar time-dependent autophagy activity after the initial suture placement and a gradual increase in autophagic flux up to 1 month after the insult. However, in our previous study using the COH model, we observed that activating autophagy immediately after the insult had a beneficial effect, promoting RGC survival after intravitreal rapamycin injection [17].

Using a retinal I/R model, Russo et al. [10] reported an early increase in autophagic activity upon retinal I/R insult, as early as 6 h after insult, which was no longer detectable after 24 h of reperfusion, thus showing that autophagy activation resulted in enhanced RGC survival, both in vivo and in vitro. Here, we observed a similar pattern using the double circumlimbal suture, demonstrating an early increase in autophagy activity. Expression levels of LC3-II, Beclin-1, and ATG family proteins were increased as early as 1 h after reperfusion, which decreased over the period of 24 to 48 h after the injury. Crucially, we observed that while LC3-II increased early (1–6 h), p62 levels remained relatively unchanged during this initial period and only began to significantly accumulate at 24 to 48 h. This strongly indicates that autophagic flux is progressively impaired in the later phase of I/R injury, contributing to RGC death. It should also be noted that the lack of p62 decrease despite LC3-II increase may suggest that autophagosome accumulation could result from impaired clearance or an altered synthesis/degradation balance during the early period of I/R insult.

Furthermore, intravitreal rapamycin administration before retinal I/R insult resulted in enhanced RGC survival through the modulation of autophagy. In this pre-conditioned group, rapamycin led to a decrease in the p62 level and an increase in the LC3B-II/LC3B-I ratio, suggesting that mTOR inhibition may help maintain functional autophagic flux that would otherwise be impaired by the I/R insult. By enhancing autophagic capacity before the acute stress, the cells may be primed to rapidly clear damaged organelles, thereby mitigating the severe damage that occurs during the initial period of I/R insult. This mechanism likely maximizes the inherently brief window of neuroprotective autophagy that naturally occurs immediately after the ischemic insult. However, as rapamycin may have pro-survival effects through other mechanisms, such as anti-oxidative or anti-inflammatory effects [11,37], future studies are needed to demonstrate the neuroprotective effects on RGCs using more specific agents targeting the autophagy machinery.

Our selection of time points for rapamycin injection was also guided by such early induction of autophagy upon I/R insult. The 1-day-prior time point was chosen to assess protective pre-conditioning. In line with this approach, pre-conditioning with rapamycin in the I/R model—although administered via a different route (intraperitoneally)—has been previously reported to be effective in enhancing RGC survival [10,38]. Meanwhile, the 6-h post-injury injection aimed to investigate if autophagy induction early after I/R injury had therapeutic effects on enhancing RGC survival. The minimal to no benefit observed in eyes treated with rapamycin at 6 h post-injury suggests that the therapeutic window for enhancing the endogenous response may be very narrow. Therefore, further investigations are warranted to precisely define the optimal timing of autophagy induction required to achieve effective neuroprotection against retinal I/R injury.

Various signaling pathways may suppress autophagy. The mTOR pathway may inhibit autophagy through the phosphorylation of the ULK1 and 4EBP1 complex [39]. Several previous studies have shown its inverse correlation with autophagy activity in glaucomatous animal models [10,17]. In accordance with previous reports, in our study, the expression levels of p-mTOR, p-ULK1, and p-4EBP1 were significantly increased in the later period after retinal I/R induction, which inversely correlated with the results observed for autophagy markers. These results further support our findings that mTOR pathway inhibition through intravitreal rapamycin injection prior to retinal I/R injury promotes autophagy activity, as confirmed by WB analyses, which eventually leads to increased RGC survival. Our findings also indicate that enhancement of autophagy activity at different time points may have varied effects on RGC survival in retinal I/R conditions.

We acknowledge that a definitive assessment of autophagic flux requires experiments using lysosomal inhibitors, such as bafilomycin A1 or chloroquine, to compare LC3-II and p62 levels in the presence and absence of such inhibitors. The lack of these flux assays is a limitation of the current study. However, the observed reciprocal relationship between early ATG protein elevation and late p62 accumulation provides strong indirect evidence of a biphasic autophagy response—initial induction followed by later failure—in this retinal I/R model. Future studies using specific flux inhibitors and genetic modulators will be necessary to further elucidate the precise kinetics of the autophagic process.

In conclusion, we established a reliable acute retinal I/R model and demonstrated that injury triggers a biphasic autophagy response regulated by the mTOR and AMPK pathways. While early autophagy serves as a natural defense, it is rapidly impaired as injury progresses. Crucially, autophagic pre-conditioning via mTOR inhibition with rapamycin significantly enhances RGC survival by priming the cellular degradative machinery before the peak of ischemic stress. These findings suggest that targeting the mTOR-mediated autophagy window is a promising strategy for treating acute glaucoma and ischemic retinal diseases.

## 4. Materials and Methods

### 4.1. Animals

Male Sprague-Dawley rats aged 7 to 8 weeks (Orient Bio, Seongnam, Republic of Korea) were used in the study. They were housed under standard conditions in a 12-/12 h light/dark cycle with food and water ad libitum. All animal care and experimental procedures complied with the ARVO statement on the Use of Animals in Ophthalmic and Vision Research, and the study was approved by the Institutional Animal Care and Use Committee of Soonchunhyang University Bucheon Hospital (approval no. SCHBCA201708). Deep sedation was achieved via intraperitoneal injection of a mixture of 40 mg/kg zolazepam/tiletamine (Zoletil; Virbac, Carros, France) and 5 mg/kg xylazine (Rompun; Bayer, Leverkusen, Germany) prior to any surgery.

### 4.2. Double Circumlimbal Suturing

Animals were divided into I/R and non-I/R (control) groups. In the I/R group, we placed double circumlimbal sutures, thus modifying the single circumlimbal suture technique to induce COH [20,21]. Briefly, single circumferential suturing (7/0 nylon) was performed around the globe approximately 0.5 to 1.0 mm behind the limbus, and another suture was placed 0.5 to 1.0 mm behind the first suture. The contralateral eye was left untreated. After 2 h of double circumlimbal suturing, both sutures were released and removed from the eyeball to induce reperfusion. The anterior segments were photographed before and immediately after single and double suturing.

### 4.3. IOP Measurements and Fundus Photography

Baseline IOP was measured using a rebound tonometer (TonoLab; iCare, Helsinki, Finland) between 11 a.m. and noon to exclude any effects of diurnal variation. IOP was measured immediately after double circumlimbal suturing and at 1 and 2 h later, as well as at immediately after suture release under anesthesia. IOP was again measured at 12 h, and at 1, 2, 3, 4, 5, 6, and 7 days after release of sutures in the awake condition without topical anesthesia. Fundus photographs (Eyemera; IIScience, Busan, Republic of Korea) were taken under sedation to evaluate retinal I/R status after suturing and suture release.

### 4.4. Intravitreal Administration of Rapamycin

Intravitreal rapamycin (37094; Sigma-Aldrich, St. Louis, MO, USA) or 0.1% DMSO injection was performed as previously described [17,40]. Briefly, after deep sedation and pupil dilation with a mixture of 0.5% tropicamide and 0.5% phenylephrine hydrochloride (Tropherine Eye Drops; Hanmi Pharm., Seoul, Republic of Korea), a sclerotomy was created using a 30-gauge needle at approximately 0.5 to 1.0 mm posterior to the limbus under visualization with a surgical microscope. Next, 2 µL rapamycin (50 ng/µL) dissolved in DMSO or 0.1% DMSO as a vehicle control was injected intravitreally using a NanoFil syringe with a blunt 35-G needle (World Precision Instruments, Sarasota, FL, USA) through the sclerotomy site. Intravitreal DMSO or rapamycin injection was conducted in eyes under anesthesia 1 day before double circumlimbal suturing, and at 6 and 24 h after the surgery (*N* = 6 for each group).

### 4.5. Tissue Preparation

Ocular tissues were processed as described previously [41,42]. Briefly, rats were deeply anesthetized and intracardially perfused with 0.1 M phosphate buffer (PB) with 150 U/mL heparin, followed by perfusion with 4% paraformaldehyde (PFA) in 0.1 M phosphate-buffered saline. The eyes were enucleated, and a 360° sclerotomy was performed around the limbus to obtain the posterior eyeball segment. Tissues were fixed in 4% PFA, followed by overnight incubation in 30% sucrose in PB and embedding in the Optimal Cutting Temperature compound. Serial sections 10 µm in thickness were mounted on adhesive slides (Histobond; Marienfeld-Superior, Lauda-Königshofen, Germany). To prepare whole retinal mounts, the posterior eyecups were fixed in 4% PFA in 0.1 M phosphate-buffered saline and flattened after creating four equidistant cuts.

### 4.6. IHC and TUNEL Staining

Retinal sections were permeabilized in 0.1% Triton X-100 with 5% goat serum for 1 h, followed by overnight incubation at 4 °C with the primary antibodies listed in Table 1. The terminal deoxynucleotidyl transferase TUNEL assay was performed as suggested by the manufacturer (catalog no. 12156792910; in situ Cell Death Detection Kit; Roche Diagnostics, Basel, Switzerland). The sections were washed and incubated for 1 h at room temperature with secondary antibodies (Alexa Fluor 488-conjugated donkey anti-rabbit IgG [Invitrogen, Carlsbad, CA, USA] and Alexa Fluor 568-conjugated donkey anti-mouse IgG [Invitrogen]). Nuclei were counterstained with 4′6-diamidino-2-phenylindole dihydrochloride (DAPI, 0.1 mg/mL; Sigma-Aldrich) for 3 min. Negative control images without primary antibodies are shown in Supplementary Figure S2. Retinal whole mounts were similarly stained; BRN-3a (MAB1585; Chemicon, Temecula, CA, USA) served as the primary antibody. Confocal microscopy (LSM510 Meta; Carl Zeiss, Jena, Germany) was used to examine and photograph the samples. RGCs were counted in eight peripheral (approximately 3 mm from the optic disc), five mid-region (2 mm from the optic disc), and three central regions (within 1 mm from the optic disc) of the whole mounts. ImageJ software (Version 1.53v, National Institutes of Health, Bethesda, MD, USA) was used for RGC counting.

### 4.7. WB Analyses

Excised retinas were lysed in 80 µL of RIPA II lysis buffer (Gendepot, Barker, TX, USA) containing Xpert phosphatase inhibitor cocktail (Gendepot, Barker, TX, USA) and Xpert protease inhibitor cocktail (Gendepot, Barker, TX, USA). Next, the lysates were centrifuged for 15 min at 4 °C at 10,000× *g*, and the supernatants were collected and assayed for protein concentration using a protein assay kit (BCA Protein Assay Kit; Thermo Scientific Inc., Waltham, MA, USA). An equal amount of total protein (20 μg) was extracted via sodium dodecyl sulfate polyacrylamide gel electrophoresis and transferred onto nitrocellulose membranes. Membranes were blocked for 1 h at room temperature using 5% skim milk, and incubations with the primary antibodies listed in Table 1 were performed overnight. After incubation with the primary antibodies, the membranes were incubated with species-specific horseradish peroxidase-conjugated goat IgG secondary antibodies (1:5000; Santa Cruz Biotechnology, Santa Cruz, CA, USA) for 1 h at room temperature. Band signals were detected using an enhanced chemiluminescence system (Bio-Rad Laboratories, Hercules, CA, USA), and the films were scanned and digitized. Band intensities were quantified using ImageJ software.

### 4.8. Transmission Electron Microscopy

One week after double circumlimbal suturing, the rats were sacrificed and the eyes were enucleated. The optic nerves were separated from the posterior eyecups and fixed in Karnovsky fixative (2% glutaraldehyde, 2% PFA, and 0.5% calcium chloride in 0.1 M PB, pH 7.4) for 12 h at 4 °C. Then the excised optic nerves were washed with 0.1 M PB for 2 h, fixed in 1% osmium tetroxide in 0.1 M PB for 1 h at room temperature, dehydrated in ethanol, embedded in Epon 812-propylene oxide, and sectioned (80 nm thick) using a Reichert Ultracut S Ultratome (Leica, Wetzlar, Germany). The sections were stained with 6% uranyl acetate and lead citrate and examined under a JEM-1011 transmission electron microscope (JEOL, Ltd., Tokyo, Japan). Images were obtained using a Morada digital camera.

### 4.9. Statistical Analyses

A quantitative comparison of the number of RGCs between control and I/R eyes was performed using ImageJ software. The Kruskal–Wallis test with post hoc analysis was used to determine statistically significant differences among the groups. All data are shown as mean ± standard error, and a Student’s *t*-test was used to identify statistical significance between groups. Statistical analyses were conducted using SPSS for Windows software (version 20.0; SPSS Inc., Chicago, IL, USA). At least three biological replicates were used for statistical analyses. Differences at *p* less than 0.05 were considered statistically significant.

## 5. Conclusions

We demonstrated a novel and simple method to induce retinal I/R using double circumlimbal suturing. Using this model, we showed that autophagy was activated in the early period after reperfusion injury, as early as 1 h after the insult, and it was depleted 1 week after the injury. Increased autophagic flux through rapamycin before the insult resulted in enhanced RGC survival. Our results suggest that targeting autophagy in glaucomatous optic neuropathy may be another potential therapeutic strategy to promote RGC survival.

## Figures and Tables

**Table 1:** Primary antibodies used in this study.

Target	Supplier and Catalog No.	Method and Dilution
LC3b	Novus Biologicals, NB100-2220 (Centennial, CO, USA)	IF 1/200, WB 1/1000
BRN-3a	Chemicon, MAB1585 (Rolling Meadows, IL, USA)	IF 1/100
NeuN	Chemicon, MAB377	IF 1/1000
p-mTOR	Abcam, ab109268 (Cambridge, UK)	WB 1/1000
ATG7	Abcam, ab223365	WB 1/1000
Beclin-1	Cell Signaling, 3495	WB 1/1000
4EBP1	Cell Signaling, 9644	WB 1/1000
p-4EBP1 (Thr37/46)	Cell Signaling, 2855	WB 1/1000
ULK1	Cell Signaling, 8054	WB 1/1000
p-ULK1 (Ser757)	Cell Signaling, 14202S	WB 1/1000
AMPK	Cell Signaling, 2603	WB 1/1000
p-AMPK (Thr172)	Cell Signaling, 2535	WB 1/1000
p62	Sigma, P0067 (Burlington, MA, USA)	WB 1/1000

p-mTOR: phosphorylated mammalian target of rapamycin; 4EBP1: 4E-binding protein 1; ULK1: Unc51-like kinase 1; AMPK: AMP-activated kinase; IF: immunofluorescence; WB: Western blotting.

## Data Availability

The original contributions presented in this study are included in the article/Supplementary Materials. Further inquiries can be directed to the corresponding author.

## Supplementary Materials

The following supporting information can be downloaded at: https://www.mdpi.com/article/10.3390/ijms27021031/s1.
