# Supplementary material for: Enhanced Retinal Ganglion Cell Survival via Autophagy Activation in a Novel Retinal Ischemia/Reperfusion Rat Model
Source: Int J Mol Sci. 2026 Jan 20;27(2):1031. doi: 10.3390/ijms27021031 (PMC12841774; doi:10.3390/ijms27021031)
Supplement: Supplementary file 1 [file ijms-27-01031-s001.zip › ijms-3934806-supplementary.pdf]

Supplemetary Materials

Enhanced Retinal Ganglion Cell Survival via Autophagy Acti-vation in a Novel Retinal Ischemia/Reperfusion Rat Model

Si Hyung Lee, Jung Woo Han, Su-ah Yoon, Hun Soo Chang and Tae Kwann Park.

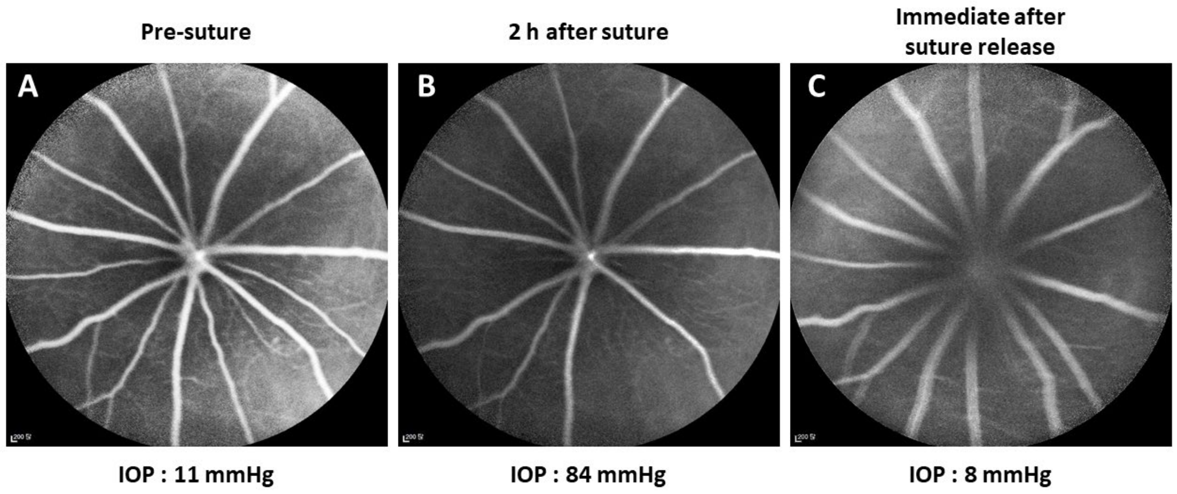

Supplementary Figure S1. Fluorescence angiography images of a rat retina (A) before, 2h after double-circumlibal suturing (B), and immediately after suture release (C).

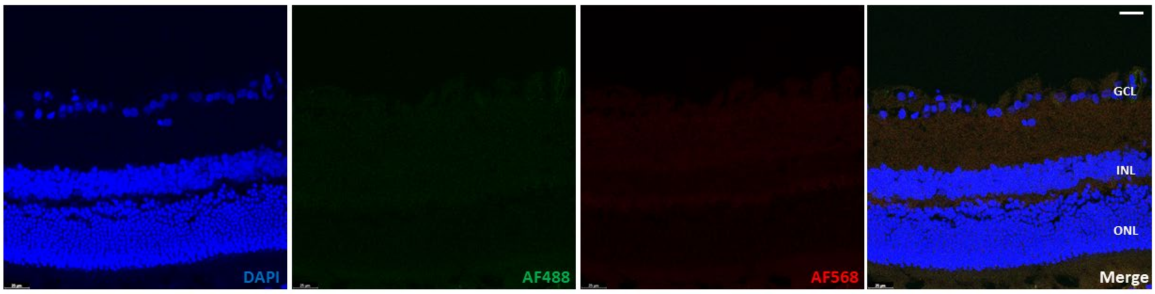

Supplementary Figure S2. A negative control image of retinal cross section without primary antibodies.
